# Population genomics identifies genetic structure and admixture in the endangered Beale’s Eyed Turtle (*Sacalia bealei*), and implications for aquatic ecology and ex situ breeding

**DOI:** 10.1007/s42995-025-00340-7

**Published:** 2026-03-03

**Authors:** Wing-Him Lee, Yik-Hei Sung, Bi Wei Low, Jonathan J. Fong

**Affiliations:** 1 Division of Science, Wu Jieh Yee School of Interdisciplinary Studies, Lingnan University, Hong Kong, SAR 999077 China; 2 School of Health, Sciences and Society, University of Suffolk, 19 Neptune Quay, Ipswich, IP4 IQJ UK; 3 Department of Biological Sciences, National University of Singapore, 16 Science Drive 4, Singapore, 117558 Singapore

**Keywords:** Conservation genomics, ddRADseq, Ex situ conservation, Population structure, *Sacalia bealei*

## Abstract

Freshwater turtles are one of the most threatened animal groups in the world, especially in Asia. The Beale’s Eyed Turtle (*Sacalia bealei*) is a highly endangered Chinese species at risk of extinction due to overexploitation for the food and pet trades. Nonetheless, Hong Kong still houses a handful of relatively healthy populations of this species, providing an increasingly rare opportunity to study the population genetics of wild populations and, in turn, guide conservation action. In this study, we used genome-wide double-digest restriction-site associated DNA sequencing (ddRADseq) markers to examine the population structure of *S. bealei* from all known wild individuals with samples suitable for DNA sequencing, representing a subset of its distribution in Hong Kong (two localities: HK1, HK2) and Fujian Province. Although genetic diversity is relatively low, we recovered four genetic clusters, with three corresponding to each of the three known localities (HK1, HK2, Fujian), and admixture between the clusters. Captive-breeding colony individuals showed mixed geographic origins, with approximately half clustering with Fujian populations and half with Hong Kong populations. These data were used to evaluate the genetic diversity and infer the geographic origin of an ex situ breeding colony with individuals of unknown provenance. These results provide important baseline information on the population structure of wild *S. bealei* and the potential geographic origins of captive turtles, which directly contribute to in situ and ex situ conservation efforts of the species.

**Supplementary Information:**

The online version contains supplementary material available at 10.1007/s42995-025-00340-7.

## Introduction

Freshwater turtles have faced a significant risk of extinction over the last few decades due to habitat degradation and over-exploitation for food and pet trades (van Dijk 2000). The Beale’s Eyed Turtle (*Sacalia bealei*), endemic to southern China, is one of the most threatened turtle species in the world (Turtle Taxonomy Working Group 2021). Conservation of this species is urgently needed due to overharvesting for the food and pet trades (Cheung and Dudgeon 2006; Sung and Fong 2018). Unabated population declines may further exacerbate the situation for endangered species, as population bottlenecks can lead to a loss of genetic diversity, thereby speeding up the extinction process (Frankham 2005). Although there have been previous genetic studies of the genus *Sacalia* (Lin et al. 2022; Shi et al. 2008), the focus has been on the four-eyed turtle (*Sacalia quadriocellata*).

*Sacalia bealei* is endemic to southern China (Guangdong, Fujian, Hong Kong and Jiangxi) (Fong and Qiao 2010; Turtle Taxonomy Working Group 2021), and previous genetic studies suggested that it exhibits low genetic and morphological variation (Lin et al. 2022; Shi et al. 2008). This species inhabits hill streams and irrigation ditches (Lau and Shi 2000), and feeds on fruits, seeds, and a variety of invertebrates (Sung et al. 2021). Currently, this species is extremely rare across its native range due to illegal hunting (Asian Turtle Trade Working Group 2000; Cheung and Dudgeon 2006; Sung and Fong 2018) and is categorized as ‘endangered’ on the IUCN Red List of Threatened Species (Asian Turtle Trade Working Group 2000). Although this study represents the largest sampling to date of known wild individuals, we only cover Fujian (one site) and Hong Kong due to the rarity of *S. bealei*.

An ex situ breeding program has been set up for *S. bealei* by the Hong Kong Society of Herpetology Foundation (HKHERP) and Ocean Park Corporation (OP). The program aims to restore wild populations in Hong Kong through the reintroduction of captive-bred individuals. While HKHERP and OP have overcome a crucial first barrier by successfully producing offspring in captivity, the geographic origin and the genetic diversity of the breeding stock are unknown, raising concerns of inbreeding within the captive population and artificial admixture by releasing the non-native genetic stock to local populations. The introduction of non-native genotypes may disrupt the natural pattern of geographic variation and have a higher chance that the non-native genotypes are poorly adapted to the local conditions (Endler et al. 2010). As such, there is an urgent need to elucidate the genetic provenance of captive turtles, which helps identify suitable individuals and locations for release, as well as recommend pairs for future breeding to preserve native genetic structure.

In this study, we used the double digest restriction-site associated DNA sequencing (ddRADseq) method to examine the genetic diversity and population structure of *S. bealei* from Fujian Province and Hong Kong (approximately 700 km apart), as well as to infer the geographic origin and genetic diversity of individuals from an ex situ breeding colony.

## Materials and methods

### Sampling

All methods used in this study were approved and performed in accordance with the relevant guidelines and regulations of Lingnan University Research Committee (Sub-Committee on Research Ethics and Safety). Wild individuals from Hong Kong (sites HK1 and HK2) were collected using baited hoop traps following Sung et al. (2013) between March and October 2015–2022. Tissue samples (tail tips or blood) were collected, and the turtles were released at the sampling site. Permission to capture, handle, and take samples from these endangered species was approved by the Agriculture, Fisheries and Conservation Department of the Hong Kong Special Administrative Region Government, China (Permit # (94) in AF GR CON 09/50 pt. 29). Hainan Normal University provided tissue samples from wild Fujian individuals from a single site (collected 2014–2015), while HKHERP provided tissue samples from the ex situ breeding colony. All samples from the ex situ breeding colony are individuals with unknown geographic origin that were donated by the public. Although some offspring have been produced from the breeding colony, only breeding stock were included in this study. In total, 132 samples were obtained (75 samples from Hong Kong, 20 samples from Fujian, and 37 samples from HKHERP). The samples from Hong Kong and Fujian cover all the wild samples known and available to us for DNA analysis. Although this is currently the largest dataset of wild *S. bealei*, there are sampling gaps (*e.g.*, Guangdong Province, Jiangxi Provice, ahead of and only one site in Fujian Province) that limits our ability to detect population structure. The exact localities of the wild samples are not disclosed to protect remaining populations of this endangered species from poaching. Samples from the ex situ breeding colony are hereafter referred to as HKHERP. Sampling locations were separated by substantial distances: Fujian and Hong Kong samples are approximately 700 km apart, while HK1 and HK2 sites are approximately 4 km apart but in different watersheds with many potential barriers to animal movement (mountain valleys, roads, reservoirs).

### Library preparation

Genomic DNA was extracted from blood or muscle samples using the Qiagen DNeasy Blood and Tissue Kit (Qiagen, Germany) following the manufacturer’s protocol. We quantified DNA using a Qubit 2.0 fluorometer (Thermo Fisher Scientific, Waltham, MA, USA) and Qubit dsDNA BR Assay Kit, and checked quality by running DNA on a 1.5% agarose gel and visualizing under UV light. The ddRADseq method was performed following Peterson et al. (2012). In summary, the restriction enzymes *SbfI* (restriction site 5′-CCTGCAGG-3′) and *MspI* (restriction site 5′-CCGG-3′) (New England Biolabs Inc.) were used to digest the DNA samples for 4 h at 37 °C. The successful digestion of samples was confirmed by running a 1.5% agarose gel and visualizing it with UV light. Serapure beads were made with SpeedBead Magnetic Carboxylate (GE Healthcare UK Limited) to purify DNA fragments. Samples were ligated with eight barcoded adaptors using T4 DNA ligase (NEB) for 30 min at 37 °C. The ligase was then inactivated at 65 °C for 10 min. The libraries were size selected between 250 and 500 bp using Serapure beads (Baym et al. 2015) and standardized to a concentration of 20 ng in 10 µL per sample for PCR amplification. The samples were amplified for 10 cycles using a Phusion polymerase High-Fidelity PCR Kit (NEB) with Illumina-indexed primers. Each individual library was uniquely labelled using a combination of eight barcoded adaptors and 12 Illumina-indexed primers, then pooled for sequencing. The samples were sequenced using a partial lane of Illumina HiSeqX PE150.

### SNP identification

Samples were first demultiplexed using unique barcode sequences and trimmed of restriction site overhangs. We then assembled loci using Stacks v2.61 (Catchen et al. 2013) denovo_map.pl pipeline, which builds loci de novo in each sample, creates a catalogue of loci for specified populations, and matches up loci across samples. Two parameters were tested to understand their impact on the results: distance allowed between stacks (*M*), distance between catalogue loci (*n*). Two combinations were tested (*M* = 3, *n* = 3; *M* = 5, *n* = 5). Since the results were similar (Supplementary Fig. S1), we use *M* = 5, *n* = 5 for the analyses. After identifying the catalogue loci and matches, we ran the “populations” program in Stacks (Catchen et al. 2013), setting a minimum minor allele count of 3 (Rivera-Colón and Catchen 2021), a minimum of 50% of individuals in a population required to process a locus for that population and calculating if the individual loci are in Hardy–Weinberg equilibrium.

Of the 132 samples, 32 were excluded due to insufficient DNA concentration for ddRADseq, and 25 were excluded due to high amounts of missing data and low sequencing coverage. Most of the removed samples included localities otherwise represented in the datasets. As such, the final sampling included 75 individuals. We constructed two datasets of individuals with > 10 × coverage for analysis (Supplementary Tables S1, S2): a subset of 42 individuals (Fujian [*n* = 13], HK1 [*n* = 16], and HK2 [*n* = 13]) to examine the population structure of wild populations, and a complete set of 75 individuals (including both wild [*n* = 42] and HKHERP individuals [*n* = 33]) to examine the geographic origin of the ex situ breeding colony.

For the two sets of individuals (42 individuals, 75 individuals), we generated two different SNP datasets—unlinked SNPs (42-SingleSNP, 75-SingleSNP) and linked SNPs (42-FullSNP, 75-FullSNP). The unlinked datasets (42-SingleSNP, 75-SingleSNP) were used for calculating the pairwise differentiation, genetic diversity and effective population size; examining the genetic clustering; and conducting hybridization analyses, while the linked datasets (42-FullSNP, 75-FullSNP) were used for the co-ancestry analysis. The linked SNP datasets are used because the analysis infers recent coalescence using haplotype linkage information to derive the co-ancestry matrix. The unlinked datasets (42-SingleSNP, 75-SingleSNP) were generated by randomly selecting 5000 SNPs that are in Hardy–Weinberg equilibrium, and reran the “populations” program to select the first SNP per locus using the “write-single-SNP” option. Individuals with > 50% missing loci were then removed using Plink v1.90 (Purcell et al. 2007). A total of 5000 SNPs were retained for these datasets. The two datasets of linked SNPs (42-FullSNP, 75-FullSNP) were generated using the “populations” program, specifying a minimum of 50% of individuals in a population required to process a locus for that population (“min-samples-per-pop” option) and filtering data by haplotypes using the “filter-haplotype-wise” option.

### Pairwise differentiation

As a preliminary analysis to examine the genetic distance between localities, the 42-SingleSNP dataset was used to calculate the genetic differences between localities based on pairwise differentiation *F*_ST_ values using GENODIVE v3.06 (Meirmans 2020). A total of 999 permutations were used to assess the significance (*P* values) of all pairwise measures.

### Genetic diversity

To understand the genetic diversity of the wild populations and the current ex situ breeding colony, the 75-SingleSNP dataset was used to calculate the genetic diversity of the complete dataset and for each of the four groups (three localities and one ex situ breeding colony) using GENODIVE v3.06 (Meirmans 2020). We calculated the observed heterozygosity (*H*_o_), expected heterozygosity (*H*_e_), total heterozygosity (*H*_t_), and inbreeding coefficient (*F*_is_). Standard deviation and 95% confidence intervals were calculated for each statistic by bootstrapping.

### Effective population sizes

To estimate contemporary effective population size (*N*_e_), the 75-SingleSNP dataset was used to calculate the effective population size using the bias-corrected measure of linkage disequilibrium (Waples and Do 2010), as implemented in NeEstimator v.2.1 (Do et al. 2014). We estimated *N*_e_ for all wild populations using a minimum allele frequency of 0.05 and 95% confidence intervals. HKHERP was excluded because it is a breeding stock, not a breeding population.

### Genetic clustering

To identify how many genetic clusters were present across localities (42-SingleSNP) and to infer the genetic provenance of HKHERP individuals (75-SingleSNP), both 42-SingleSNP and 75-SingleSNP datasets were used for PCA and Bayesian clustering analyses. The *snpgdsPCA* function from the R package *SNPRelate* v1.34.1 (Zheng et al. 2012) was first used to visualize the genetic clustering on a PCA. Subsequently, the Bayesian clustering analysis method implemented in STRUCTURE v2.3.4 (Pritchard et al. 2000) was used to infer the number of genetic clusters (K). We ran 20 replicates for *K* values of 1–10 with 100,000 burn-in and 200,000 MCMC iterations. STRUCTURE HARVESTER v0.6.94 (Earl and von Holdt 2012) was used to infer the uppermost level of population structure using the Evanno method (Evanno et al. 2005), but we also visually compared clustering patterns at different *K* values (Janes et al. 2017). STRUCTURE runs were summarized and visualized using CLUMPAK (Kopelman et al. 2015).

### Co-ancestry analysis

In addition to genetic clustering, we want to examine the co-ancestry between localities (42-FullSNP) and compare between wild and HKHERP individuals (75-FullSNP). Therefore, both 42-FullSNP and 75-FullSNP datasets were used to infer recent co-ancestry using the package fineRADstructure (Lawson et al. 2012; Malinsky et al. 2018). This approach is seen as complementary to PCA and STRUCTURE analyses. We first used RADpainter (Malinsky et al. 2018) to infer recent ancestral relationships among individuals (the “co-ancestry matrix”), followed by the fineSTRUCTURE algorithm (Lawson et al. 2012) to assign individuals to populations using 100,000 MCMC iterations with a burn-in of 100,000, and build a tree using 100,000 MCMC iterations. It should be noted that this tree represents the genetic similarity between populations, and not a phylogenetic tree. The results were visualized using the R scripts fineradstructureplot.r and finestructurelibrary.r (Malinsky et al. 2018).

### Hybridization analysis

To assign individuals to known gene pools and detect hybrids, we conducted hybridization analyses on the 75-SingleSNP dataset (comprising all study individuals) using the R package HYBRIDDETECTIVE v0.1.0.9000 (Wringe et al. 2017) and NEWHYBRIDS v1.1 (Anderson and Thompson 2002).

For HYBRIDDETECTIVE, we first tested the efficacy of diagnostic SNP panels at identifying simulated multigenerational hybrids. The *getTopLoc* function was used to extract panels of 100, 200, 300, and 400 SNPs with the highest overall *F*_ST_ and not in linkage disequilibrium (*R*^2^ < 0.2). We then used the *freqbasedsim_AlleleSample* function to generate two replicates of three simulated datasets of multigenerational hybrids based on genotype frequencies of wild individuals with > 90% STRUCTURE assignment to the groups being compared (*i.e.*, Hong Kong vs. Fujian, HK1 vs. HK2). Six multigenerational hybrid classes were generated—pure individuals of both parent populations (Pure1 and Pure2), F1 and F2 hybrids, and backcrosses to each parent population (BC1 and BC2). The posterior probabilities of simulated individuals belonging to the six hybrid classes were estimated using NEWHYBRIDS with 50,000 burn-in and 200,000 MCMC sweeps. The accuracy and efficiency with which simulated hybrids could be correctly identified using different SNP panel sizes were assessed using the *hybridPowerComp* function in HYBRIDDETECTIVE.

We subsequently applied diagnostic SNP panels to experimental (non-simulated) individuals for hybrid detection. SNP panels with the greatest hybrid detection power (based on our previous simulation tests) were subsetted from experimental individuals. Additionally, we combined this experimental dataset with pure individuals from the simulated dataset (Pure1 and Pure2) using the *nh_analysis_data_generateR* function to improve assignment power and help with Markov chain convergence (Anderson and Thompson 2002; Wringe et al. 2017), and specified prior information on simulated individuals using the ‘z’ (known genotype assignment) and ‘s’ (to be sampled separately from mixture of interest) options. We then inferred genetic assignment of experimental individuals to hybrid classes using NEWHYBRIDS with 50,000 burn-in and 200,000 MCMC sweeps.

Since NEWHYBRIDS can only detect hybridization between two known source populations at any one time, we carried out two sequential rounds of analyses: (1) testing for hybridization between Hong Kong (HK1 and HK2 combined) and Fujian genotypes, using all 75 wild and captive individuals, and (2) testing for hybridization between HK1 and HK2 genotypes, including only individuals that were assigned as pure Hong Kong genotypes in the previous round.

## Results

### Dataset details

The number of reads varied across individuals for the subset of 42 individuals (595,944–4,069,205; average = 1,900,171) (Supplementary Table S1) and the complete set of 75 individuals (565,619–3,879,567; average = 1,486,789) (Supplementary Table S2). For the two datasets of linked SNPs, a total of 94,782 SNPs (42-FullSNP) and 75,423 SNPs (75-FullSNP) were retained. We constructed two datasets of individuals with > 10 × coverage for analysis (Supplementary Tables S1, S2): a subset of 42 individuals (Fujian [*n* = 13], HK1 [*n* = 16], and HK2 [*n* = 13]) to examine the population structure of wild populations, and a complete set of 75 individuals (including both wild [*n* = 42] and HKHERP individuals [*n* = 33]) to examine the geographic origin of the ex situ breeding colony.

### Wild populations only (42-SingleSNP and 42-FullSNP datasets)

The pairwise differentiation *F*_ST_ values based on the 42-SingleSNP dataset were calculated for the three locations (HK1, HK2, Fujian) (Table 1). All values are statistically significant (*P* < 0.05) (Supplementary Table S3). The *F*_ST_ values are generally low in all cases, with the *F*_ST_ value between HK2 and HK1 being the lowest (0.076), and the highest between HK2 and Fujian (0.094).

**Table 1:** Pairwise differentiation *F*_ST_ value of all localities. All values are statistically significant (*P* < 0.05) (Supplementary Table S3)

	HK1	HK2	Fujian
HK1	–		
HK2	0.076	–	
Fujian	0.077	0.094	–

**Fig. 1:**
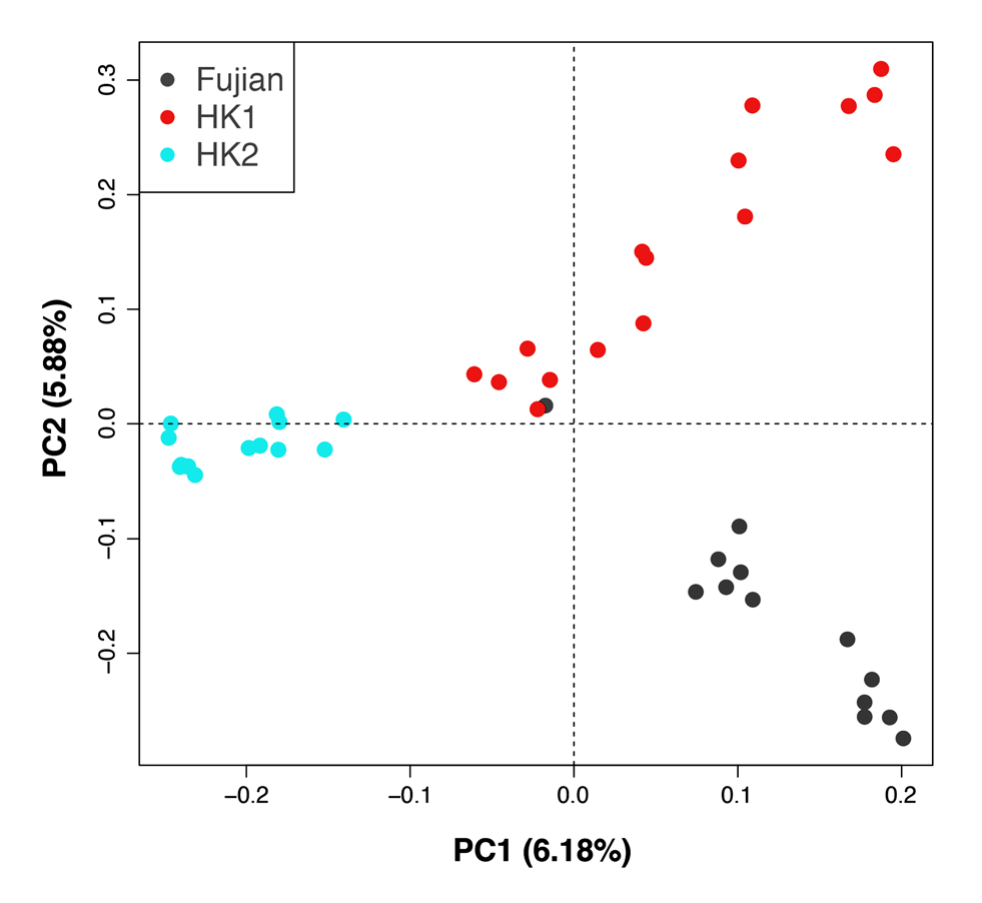
Principal component analysis (PCA) plot of *Sacalia bealei* from three localities (Fujian, HK1, HK2) using the 42-SingleSNP dataset. PCA result based on PC1 (6.18% variation) and PC2 (5.88% variation). Note the single Fujian individual clustering with HK1 samples, discussed as potential human-mediated translocation

The PCA recovered three genetic clusters that are largely separated based on location (Fig. 1). In general, PC1 (6.18% variation) separates HK2 from HK1 and Fujian, while PC2 (5.88% variation) separates HK1 and Fujian, with HK2 in between. HK1 samples are broadly scattered in both PC. Noteworthy is that a single Fujian sample (black) clusters with HK1 samples (red). The amount of variance explained by the principal components is similar to that found in another study of *Sacalia* (4.9%–6.4%) (Lin et al. 2022) and other studies using SNPs (pangolins: 4.38%–4.47%; Queen Snapper: 3.38%–6.06%) (González-García et al. 2025; Nash et al. 2018).

**Fig. 2:**
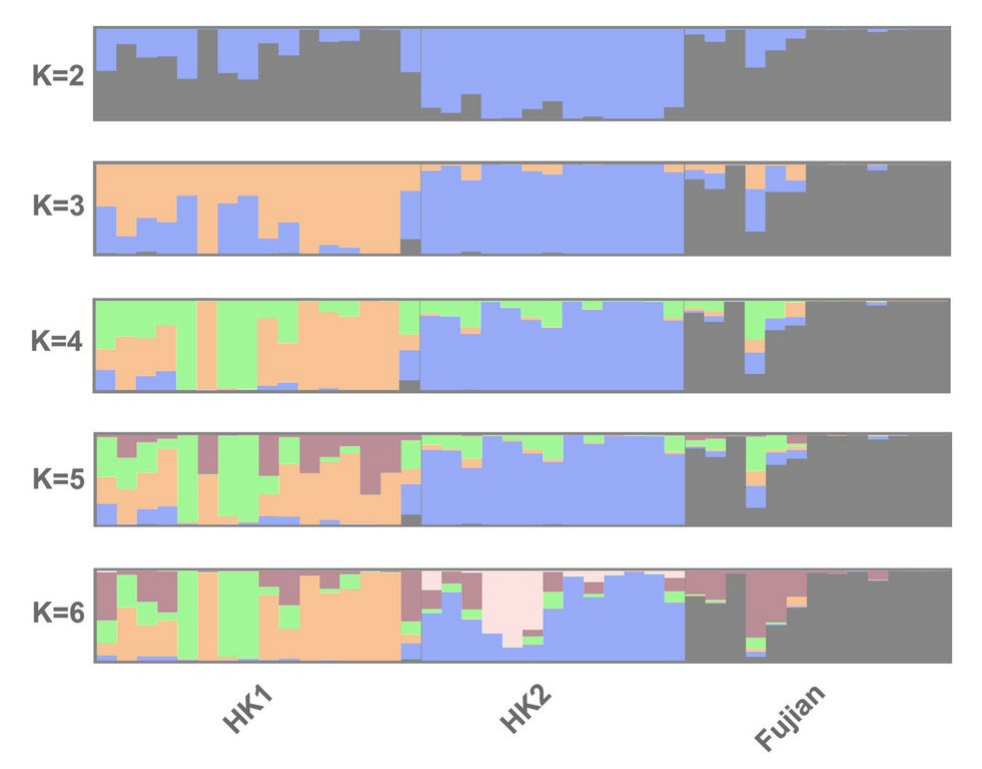
Bayesian clustering analysis (STRUCTURE) of *Sacalia bealei* from three localities (Fujian, HK1, HK2) using the 42-SingleSNP dataset, which was clustered and visualized using CLUMPAK. Each color represents a different cluster. *K* = 3 was selected as the most likely based on Δ*K* (Supplementary Fig. S2)

The STRUCTURE plots for *K* = *2* to *K* = *6* are shown in Fig. 2. The Evanno method identified *K* = 3 as the most likely *K* value (Supplementary Fig. S2). It should be noted that the uppermost level of genetic structure pattern is likely detected by Δ*K*, but further genetic sub-structuring can only be inferred by examining other *K* values (Janes et al. 2017). For both *K* = 3 and *K* = 4, the genetic clusters generally correspond to the three localities, with some admixture between localities (as seen by the mixture of colors representing individuals in the Fig. 2). As the *K* value increases, HK1 exhibits more genetic sub-structuring. In particular for *K* = 4, HK1 contains highly admixed individuals and two distinct clusters, which corresponds to the broadly scattered HK1 individuals in the PCA plot (Fig. 1). There is one Fujian individual with a similar admixture pattern to HK1, which corresponds to the Fujian individual that clusters with HK1 individuals in the PCA plot (Fig. 1). It should be noted that Fujian samples all come from a single locality, which limits our ability to detect population structure in Fujian.

**Fig. 3:**
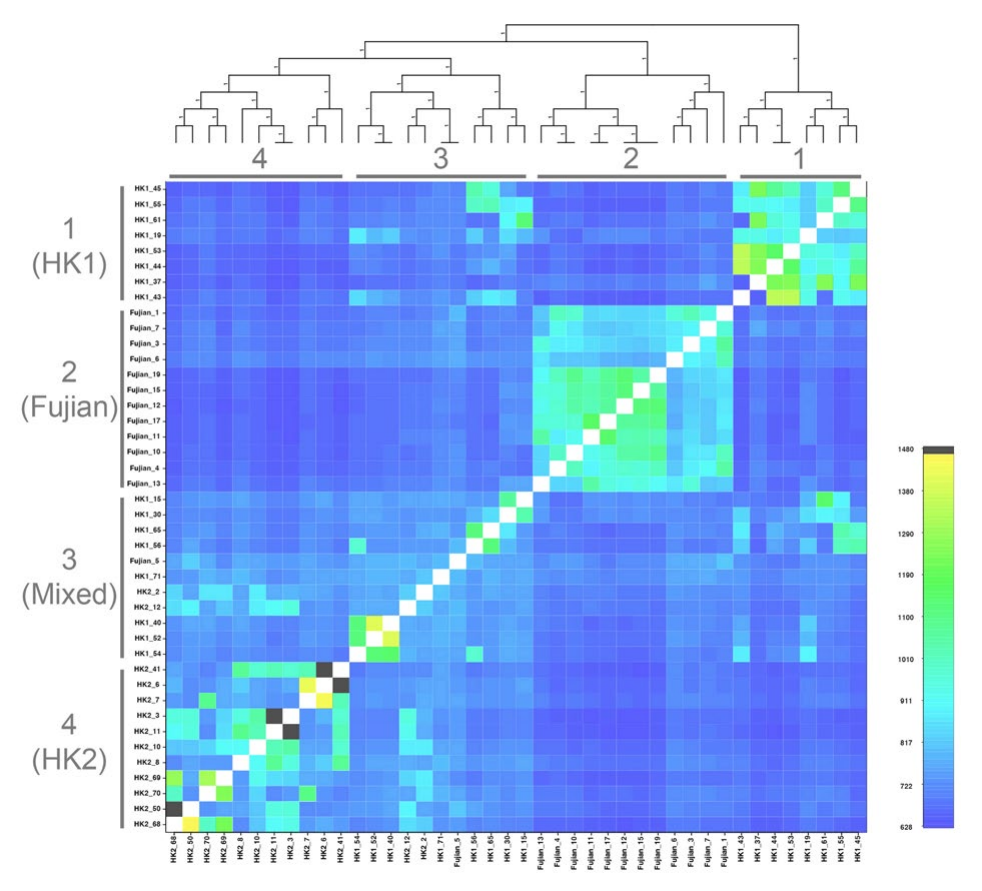
fineRADstructure co-ancestry matrix using the 42-FullSNP dataset for *Sacalia bealei*. Black/yellow cells indicate the highest level of co-ancestry, while dark blue cells indicate the lowest level of co-ancestry. The four recovered clusters are labelled with numbers (1–4)

The fineRADstructure analysis using the 42-FullSNP dataset identified four main genetic clusters (Fig. 3). Three of the clusters correspond to samples from a single locality (cluster 1 [HK1], cluster 2 [Fujian], cluster 4 [HK2]), while the remaining cluster 3 (Mixed) is comprised mostly of samples from HK1, but also includes samples from HK2 and Fujian. This result suggests that there is low but detectable population differentiation. The average co-ancestry level within cluster 3 is generally low, with some of the individuals having a higher co-ancestry relationship with individuals from the same locality in other clusters. These results correspond to the genetic structure suggested in the STRUCTURE analysis (Fig. 2) that identified two distinct clusters in HK1 (with *K* = 4) and admixture between localities. The lowest level of co-ancestry was found between individuals from cluster 2 (Fujian) and the other three clusters. The highest level of co-ancestry was found between individuals from cluster 3 (Mixed) and cluster 4 (HK2). In general, there is a high relatedness between clusters that have low *F*_ST_ values (Table 1) among the three localities, suggesting that the wild populations are closely related to each other, but the genetic distances are slightly different among localities.

### Wild and captive individuals combined (75-SingleSNP and 75-FullSNP datasets)

Summary statistics of genetic diversity across all samples are shown in Table 2. The overall genetic diversity is low among samples. Total heterozygosity (*H*_t_ = 0.176) is slightly higher than the observed heterozygosity and expected heterozygosity (*H*_o_ = 0.146; *H*_e_ = 0.167). The inbreeding coefficient is positive but close to zero (*F*_is_ = 0.131), indicating that the observed homozygotes are slightly overrepresented.

**Table 2:** Summary statistics of genetic diversity across all groups (HK1, HK2, Fujian, HKHERP) using 75-SingleSNP dataset. *H*_*o*_ observed heterozygosity, *H*_*e*_ expected heterozygosity, *H*_*t*_ total heterozygosity, *F*_*is*_ inbreeding coefficient

Statistic	Value	Std. Dev	c.i. 2.5%	c.i. 97.5%
H_o_	0.146	0.002	0.141	0.15
H_e_	0.167	0.002	0.163	0.172
H_t_	0.176	0.002	0.171	0.18
F_is_	0.131	0.005	0.121	0.14

The genetic diversity statistics for each population are shown in Table 3. Genetic diversity is similar across the three localities and HKHERP. Observed heterozygosity is highest in HK1 (*H*_o_ = 0.157) and lowest in HKHERP (*H*_o_ = 0.138), while expected heterozygosity is highest in Fujian (*H*_e_ = 0.172) and lowest in HK2 (*H*_e_ = 0.163). All values for the inbreeding coefficient are positive but close to zero, with HK1 having the lowest inbreeding coefficient (*F*_is_ = 0.048), and Fujian having the highest (*F*_is_ = 0.184). The ex situ breeding colony has a slight heterozygote deficiency (*F*_is_ = 0.181).

**Table 3:** Genetic diversity statistics and effective population sizes for each population using 75-SingleSNP dataset. *H*_*o*_ observed heterozygosity, *H*_*e*_ expected heterozygosity, *F*_*is*_ inbreeding coefficient, *N*_*e*_ effective population sizes with 95% confidence intervals

Locality/Group	*N*	*H*_o_	*H*_e_	*F*_is_	*N*_e_
HK1	16	0.157	0.165	0.048	11.8 (11.6–12)
HK2	13	0.146	0.163	0.108	14.2 (13.9–14.5)
Fujian	13	0.14	0.172	0.184	96.2 (86.7–107.9)
HKHERP	33	0.138	0.169	0.181	–

Low F_is_ values indicate minimal inbreeding, while positive values suggest slight heterozygote deficiency typical of small populations

The effective population sizes (*N*_e_) with 95% confidence intervals are shown in Table 3. The largest effective population size is detected in Fujian (*N*_e_ = 96.2), while HK1 and HK2 have small effective population sizes (*N*_e_ = 11.8; *N*_e_ = 14.2).

**Fig. 4:**
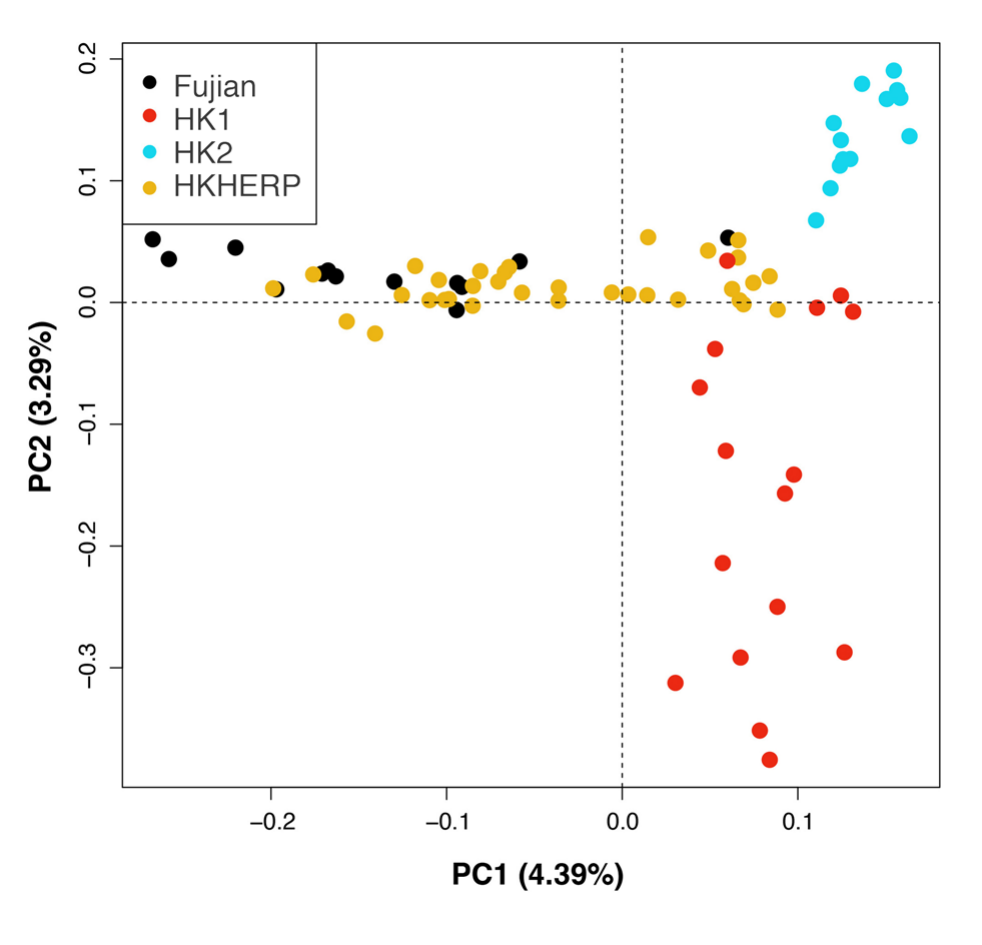
Principal component analysis (PCA) plot of *Sacalia bealei* from three localities (Fujian, HK1, HK2) and the ex situ breeding colony from Hong Kong Society of Herpetology Foundation (HKHERP) using the 75-SingleSNP dataset. PCA result based on PC1 (4.39% variation) and PC2 (3.29% variation)

The PCA plot based on the 75-SingleSNP dataset is shown in Fig. 4. Similar to the 42-SingleSNP dataset, the wild samples separate into three clusters based on locality. However, the individuals from the HKHERP breeding colony are broadly dispersed, indicating that the group is genetically diverse, and individuals likely have different origins; some individuals cluster more closely with Fujian, others more closely with HK1, and some in between Fujian and HK1. PC1 (4.39% variation) separates Fujian and Hong Kong individuals, as well as disperses HKHERP individuals, while PC2 (3.29% variation) separates HK1 and HK2.

**Fig. 5:**
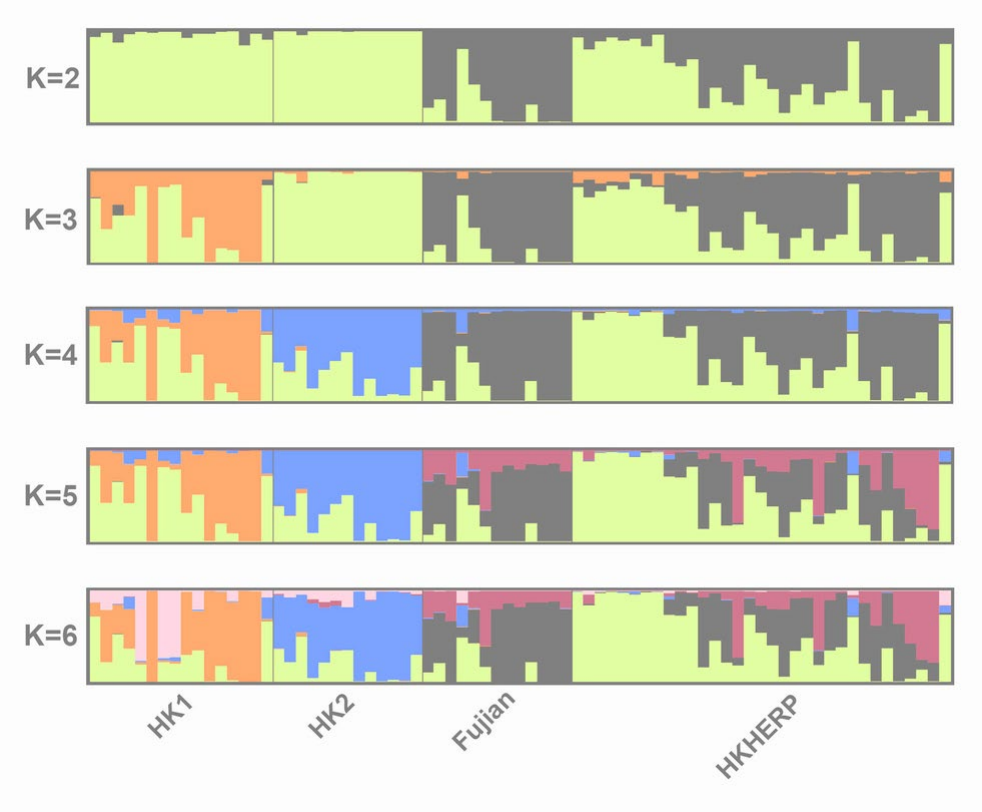
Bayesian clustering analysis (STRUCTURE) of *Sacalia bealei* from three localities (Fujian, HK1, HK2) and the ex situ breeding colony from Hong Kong Society of Herpetology Foundation (HKHERP) using the 75-SingleSNP dataset, which was clustered and visualized using CLUMPAK. Each color represents a different cluster. *K* = 2 was selected as the most likely based on Δ*K* (Supplementary Fig. S3)

The STRUCTURE results for *K* = 2 to *K* = 6 are shown in Fig. 5. The Evanno method identified *K* = 2 as the most likely *K* value (Supplementary Fig. S3). Fujian is the most distinct cluster among wild localities, while HKHERP shows high variation in clustering. For both *K* = 3 and *K* = 4, the genetic clustering of the wild localities generally corresponds to Fig. 2 (STRUCTURE analysis of only wild populations). For HKHERP at *K* = 2, around half of the individuals clustered with Fujian individuals, whereas the remainder clustered with Hong Kong populations.

**Fig. 6:**
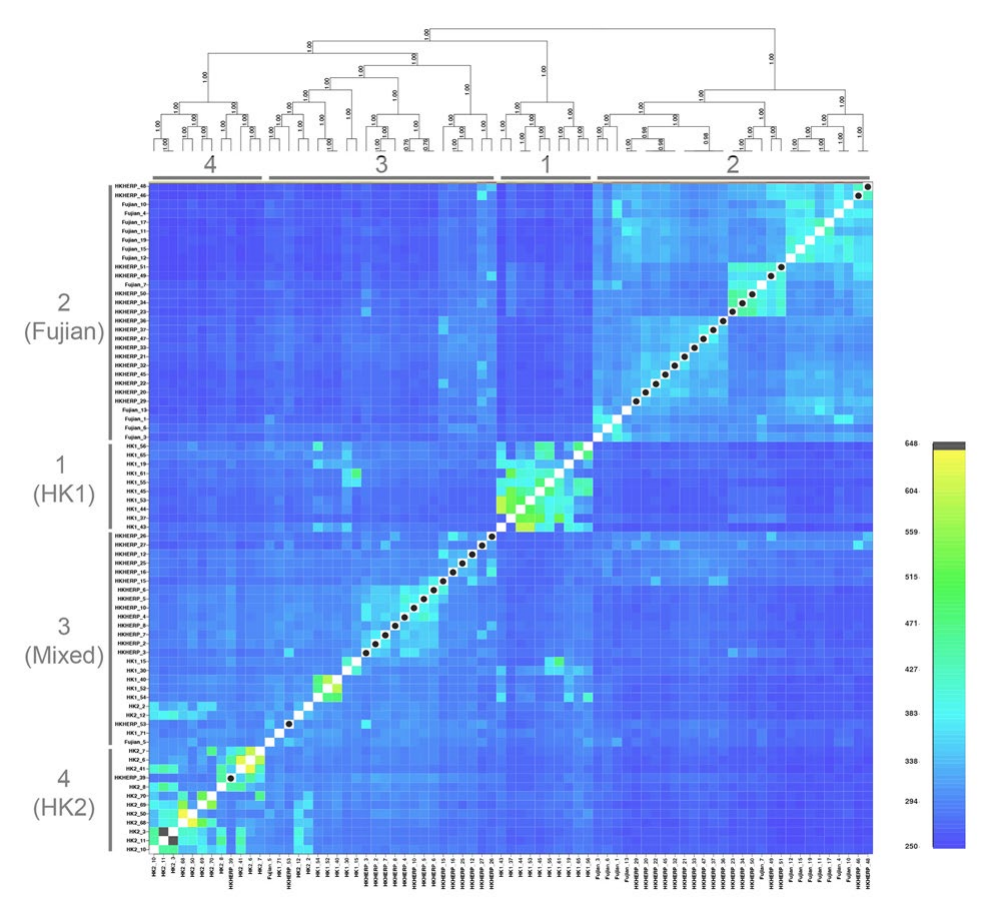
fineRADstructure co-ancestry matrix using the 75-FullSNP dataset for *Sacalia bealei*. Black/yellow cells indicate the highest level of co-ancestry, while dark blue cells indicate the lowest level of co-ancestry. The black dots in the diagonal cells indicate the ex situ breeding colony individuals from Hong Kong Society of Herpetology Foundation (HKHERP). The four recovered clusters are similar to Fig. 3, are labelled with the same numbers (1–4)

The fineRADstructure analysis using the 75-FullSNP dataset identified four main clusters (Fig. 6). The HKHERP individuals fell into three clusters: cluster 2 (Fujian, 17 individuals) and cluster 3 (Mixed, 15 individuals), while the cluster 4 (HK2, one individual). These results are similar to the PCA (Fig. 4) and STRUCTURE (Fig. 5) analyses, where around half of the HKHERP individuals cluster with the Fujian individuals and the other half had closer genetic affinities to Hong Kong populations.

The captive-breeding colony analysis revealed important findings for conservation management. Of the 33 HKHERP individuals analyzed, genetic clustering suggested diverse geographic origins. The NEWHYBRIDS analyses on simulated data (Supplementary Figs. S4, S5) found that the 400-SNP panel had the highest power (accuracy x efficiency) in detecting hybrids between Hong Kong and Fujian genotypes, correctly identifying all six hybrid classes (Pure1, Pure2, F1, F2, BC1, BC2) with a power of at least 75% at a critical posterior probability threshold of 85%. For hybrid detection between HK1 and HK2 genotypes, the 300- and 400-SNP panels performed similarly, with both correctly identifying all six hybrid classes with a power of 100%; therefore, we deemed the 300-SNP panel sufficient for hybrid detection between HK1 and HK2 genotypes.

**Fig. 7:**
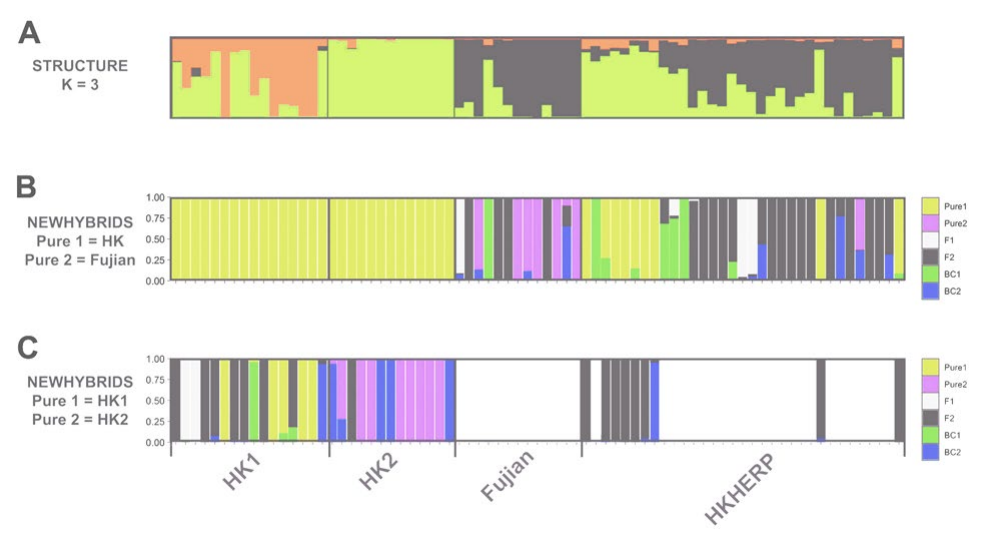
Hybrid analyses of wild-caught and captive individuals using NEWHYBRIDS. Each vertical bar represents one individual, with colored segments showing probability of assignment to hybrid categories (Pure1/Pure2 = parental types; F1 = first-generation hybrid; F2 = second-generation hybrid; BC1/BC2 = backcrosses). **A** STRUCTURE plot for *K* = 3 for comparison. **B** NEWHYBRIDS analysis of all 75 individuals, with Hong Kong (HK1 and HK2 combined) and Fujian genotypes designated as reference “pure” populations (Pure1 and Pure2, respectively). **C** second NEWHYBRIDS analysis carried out on a subset of 38 individuals assigned to 100% Hong Kong genotype in the first analysis, with HK1 and HK2 genotypes designated as reference “pure” populations (Pure1 and Pure2, respectively)

For the first round of NEWHYBRIDS analysis, with Hong Kong and Fujian genotypes as reference populations (Fig. 7B), all 29 wild-caught Hong Kong individuals were assigned as 100% Hong Kong genotype. Of the 13 wild-caught Fujian individuals, six were assigned as 100% Fujian genotype, one as a F1 hybrid, four as F2 hybrids, one as a Fujian backcross (75% Fujian genotype and 25% Hong Kong genotype), and one as a Hong Kong backcross (75% Hong Kong genotype and 25% Fujian genotype). Amongst the 33 HKHERP captive individuals, nine were assigned as 100% Hong Kong genotype, one as 100% Fujian genotype, and 23 as hybrids (two F1 hybrids, 16 F2 hybrids, four Hong Kong backcrosses, and one Fujian backcross).

The second round of NEWHYBRIDS analysis, with HK1 and HK2 genotypes as reference populations (Fig. 7C), was conducted on 38 individuals that were assigned to 100% Hong Kong genotype (29 wild and nine captive). For the 16 wild-caught HK1 individuals, five were identified as 100% HK1 genotype, two as F1 hybrids, seven as F2 hybrids, one as HK1 backcross (75% HK1 and 25% HK2), and one as HK2 backcross (75% HK2 and 25% HK1). For the 13 wild-caught HK2 individuals, eight were identified as 100% HK2 genotype, one as F2 hybrid, and as four HK2 backcrosses (75% HK2 and 25% HK1). Amongst the nine captive HKHERP individuals previously identified as 100% Hong Kong genotype, eight were identified as F2 hybrids and one as a HK2 backcross (75% HK2 and 25% HK1).

## Discussion

In this study, we used ddRADseq data to examine the population structure of *S. bealei* from Fujian Province and Hong Kong, representing the largest dataset of known wild samples, and is a major accomplishment given the rarity of *S. bealei*. The genetic diversity is relatively low for the species/localities and relatedness is high between localities, but we uncovered three main genetic clusters generally corresponding to locality, with low levels of admixture between localities. The genetic structure allows us to infer the geographic origin of the ex situ breeding colony by comparing individuals in the breeding colony (HKHERP) to wild individuals with geographical provenance. Using hybridization analyses, we genetically assign individuals to pure and hybrid categories (F1, F2, backcross), which aided in identifying appropriate individuals for captive breeding. We discuss all findings in detail below.

### Genetic diversity and population structure of wild *S. bealei*

Shi et al. (2008) and Lin et al. (2022) compared the phylogenetic relationships and population structure of *S. bealei* and its sister species (*S. quadriocellata*), using mitochondrial and nuclear genes. They found *S. quadriocellata* exhibits high genetic diversity with distinct populations, while *S. bealei* has low genetic variation and no genetic structure. In our study using ddRADseq data, we similarly found low genetic diversity in *S. bealei* (*H*_*o*_ = 0.146, *H*_*e*_ = 0.167) (Table 2), indicating potential inbreeding. These values are lower than two other endangered Asian turtle species (*Batagur trivittata*: *H*_o_ = 0.219, *H*_*e*_ = 0.213, *n* = 445; *B. affinis*: *H*_o_ = 0.213, *H*_*e*_ = 0.208, *n* = 136) (Çilingir et al. 2017, 2019), although this may be influenced by our smaller sample size (*n* = 75). These *Batagur* species are among the world’s 25 most endangered tortoises and freshwater turtles (Turtle Conservation Coalition 2011), so the lower genetic diversity underscores the dangers and conservation challenges *S. bealei* faces.

Despite low genetic diversity, we discovered *S. bealei* contains several distinct genetic clusters. Although the genetic differentiation between *S. bealei* populations is low (*F*_ST_ = 0.076–0.094) (Table 1), the values are statistically significant (Supplementary Table S3). Furthermore, all three geographic localities correspond to specific clusters in PCA (Fig. 1), STRUCTURE (Fig. 2), and co-ancestry analysis (Fig. 3), indicating genetic structure between the sampled localities, albeit with some level of gene flow between populations. The variance explained by PC1 and PC2 is relatively low (6.18% and 5.88%) (Fig. 1) possibly due to recent divergence and/or recent gene flow, supported in part by our discovery of admixture between localities. Similar patterns of low genetic diversity but with detectable genetic structure have been observed in the Bog Turtle (*Glyptemys muhlenbergii*) (Dresser et.al. 2018). In their study, although 209 samples were collected from 33 sites, the estimated number of individuals in many sampled populations is lower than 20 (Dresser et.al. 2018), which illustrates the difficulty in sampling endangered freshwater turtles.

### Comparison of individuals from Fujian Province and Hong Kong

In general, Fujian is the most genetically distinct of the three geographic locations. It shows greater similarity to HK1 (*F*_ST_ = 0.077) than to HK2 (*F*_ST_ = 0.094) (Table 1). This result mirrors the co-ancestry analysis, where the co-ancestry level between Fujian and Hong Kong samples is also low (Fig. 3). This is not surprising given the geographic distance between Fujian and Hong Kong (approximately 700 km). Noteworthy is that one Fujian individual clusters HK1 individuals (Fig. 1), which is also seen in both the STRUCTURE plot (*K* = 3) (Fig. 2), and in the cluster 3 of the co-ancestry analysis (Fig. 3). We discuss this individual in more detail below.

Of the 13 wild Fujian individuals, six were identified to be pure Fujian individuals, with the others as hybrids (one F1, four F2, one Fujian backcross, one HK backcross) (Fig. 7B). Since we have not sampled from the entire distribution of *S. bealei* (*e.g.*, Guangdong and Jiangxi Provinces), we are uncertain whether the individuals assigned to be hybrids are truly hybrids, representatives of unsampled regions, or natural genetic intermediates. However, these results suggest that the Fujian population is genetically heterogenous, with some historical gene flow with other regions, as shown by a large effective population size (*N*_e_ = 96.2) (Table 3).

Our dataset uncovered an interesting case of a single wild Fujian individual clustering with HK1 (Fig. 1). Our hybridization analysis inferred this individual to be a backcross to HK populations (75% HK, 25% Fujian). We provide two alternative hypotheses for this individual, of which the current data cannot distinguish between. First, this anomalous Fujian individual may represent natural genetic variation. The limitation of this study is that we were only able to sample wild individuals from a single site in Fujian, and these individual may represent genetic diversity from another site. Second, this individual may represent a case of relatively long-range “migration” in *S. bealei*, either as a natural genetic intermediate (possibly from Guangdong Province) moving to Fujian, or an artificial translocation of an individual from Hong Kong to Fujian Province, given its distinctiveness from other Fujian individuals, the presence of only a single individual, and the prevalence of an extensive turtle trade in southern China (Gong et al. 2009; Lau and Shi 2000). Hong Kong contains a high diversity of turtle species in food and pet markets (Cheung and Dudgeon 2006), with many wild-caught individuals exported to mainland China in the 1990s (Cheung and Dudgeon 2006). This raises the possibility that turtles collected from Hong Kong were released (intentionally or unintentionally) to the wild in mainland China. Reasons for such releases include abandonment by pet owners, religious practices, or release by the local government after confiscation. In developing countries like Vietnam, officials frequently release confiscated turtles near confiscation sites, regardless of the actual geographical provenance (Le et al. 2020). A similar situation exists in mainland China, where no regulated translocation programs are being reported, and monitoring of released animals is inadequate; the impact on wild populations is unknown (Hong et al. 2022). In Hong Kong, there are also no guidelines for releasing confiscated turtles, increasing the likelihood of non-native individuals being released.

To differentiate between the two hypotheses, more wild samples from different localities of Fujian and Guangdong Provinces are necessary. If the anomalous Fujian individual in our current study remains the only “translocated” individual, it would strongly support our hypothesis of human-mediated translocation. If more Fujian samples are found to be closely related to Hong Kong genotypes, this could represent a larger animal release, or alternative explanations of natural genetic variation or historical gene flow need to be considered.

### Relationship between HK1 and HK2

Although HK1 and HK2 have low pairwise differentiation (*F*_ST_ = 0.076), genetic clustering analyses (PCA and STRUCTURE) recover distinct clusters for HK1 and HK2. This suggests that these populations have been reproductively isolated for several generations. Several landscape features (mountain valleys and roads) may be major barriers to *S. bealei*, as they are stream-dwelling turtle species with restricted habitat selection (*e.g.*, deep stream pools, abundant stone caves, greater fruit abundance, and high benthic species diversity and abundance) (Yuan et al. 2025). The anthropogenic addition of roads may have increased isolation between the two localities. A comparable situation is seen in a study of the Eurasian Red Squirrel (*Sciurus vulgaris*), where both historical (geographical distance) and recent habitat change (deforestation) impacted gene flow and population differentiation (Trizio et al. 2005).

Additionally, reproductive rate may affect the frequency of gene flow between localities. *Sacalia bealei* has a low intrinsic reproductive rate due to three main factors. First, *S. bealei* has a relatively small clutch size, with females producing one clutch with two eggs per year (Lin et al. 2018). Second, *S. bealei* has relatively long incubation period (mean = 94.7 days) compared to other species (*i.e.,* mean = 62.25 in *Trachemys scripta elegans*) (Lin et al. 2018). Lastly, to exacerbate the situation, poaching by humans is common, as illegal traps have been encountered at both sites in Hong Kong (HK1 and HK2). The decrease in population size would make it difficult to find mates, further reducing gene flow, which could correspond to low effective population sizes in HK1 and HK2 (*N*_e_ = 11.8; *N*_e_ = 14.2) (Table 3). Although there is admixture between localities, low reproductive rate and poaching might affect the population growth. This scenario might cause HK1 and HK2 to remain as two distinct genetic groups, even with some levels of historical admixture between localities.

Despite historical isolation, admixed individuals were identified in hybridization analyses (11/16 HK1 individuals, 5/13 HK2 individuals) (Fig. 7C). Comparing the proportion of hybrids in HK1 and HK2, it seems that gene flow is asymmetrical, with more individuals moving from HK2 to HK1. This pattern could be due to natural causes (*e.g.*, elevational differences and habitat quality) that make movement in one direction easier/more appealing to turtles or artificially caused by the translocation of individuals from HK2 to HK1. Admixture between populations can increase the genetic diversity of the species, which reduces the negative consequences of inbreeding depression (Converse et al. 2017). Given the proximity of HK1 and HK2, outbreeding depression is unlikely; however, long-term genetic monitoring is necessary to assess population fitness. The movement and migration patterns of *S. bealei* are poorly studied, but so far, migration across watershed boundaries (*i.e.*, highlands bordering catchments) has not been recorded, in accordance with its highly aquatic habit. Nonetheless, more studies are needed to investigate the dispersal rate and migration ability of this species to determine the impacts of physical barriers on gene flow and understand whether the observed pattern is natural or artificial.

### Genetic relationship between the ex situ breeding colony and wild populations

Captive breeding is a common ex situ conservation approach for preserving endangered species, especially freshwater turtles (He et al. 2010; Schmidt et al. 2018; Shelmidine et al. 2016; Weissenbacher et al. 2015). HKHERP and OP started an ex situ breeding program for *S. bealei*, using individuals with unknown provenance that were donated by hobbyists and the public. Identifying the genetic variation and geographic origin of ex situ breeding colony is crucial for effective conservation and reintroduction efforts. Çilingir et al. (2017) used ddRADseq to study the critically endangered Burmese Roofed Turtle (*B. trivittata*), ensuring genetic diversity and minimizing inbreeding risks, to establish a captive assurance population. In contrast, the current ex situ breeding program of *S. bealei* is not genetically managed, raising a concern of either inbreeding or genetic pollution in native gene pools upon reintroduction, which could disrupt the genetic structure of wild populations (Laikre et al. 2010).

Given the distinct population structuring in wild *S. bealei* populations, we can infer the geographical origin of individuals in the ex situ breeding colony through a variety of analyses. Both PCA (Fig. 4) and co-ancestry analysis (Fig. 6) show that HKHERP individuals are broadly scattered, indicating that individuals likely originate from a mix of localities, although a significant proportion of HKHERP individuals cluster with those from Fujian in these analyses. Of the 33 HKHERP individuals, the co-ancestry analysis identifies one individual in the HK2 cluster, 17 in the Fujian cluster, and the remaining 15 individuals unknown as they are nested in cluster 3 with mixed localities. Our results suggest that the HKHERP breeding colony has individuals that could help augment both Fujian and Hong Kong populations.

The hybridization analysis, using a Bayesian model-based clustering framework, recovered a different result. Only one pure individual was identified from Fujian, with the remaining 32 individuals identified as various hybrids (Fig. 7B, C). Pessimistically, these results seem to indicate that the existing breeding colony cannot be used to augment Hong Kong populations due to the lack of pure Hong Kong individuals. However, admixture was already identified within Hong Kong populations. In such a case, mixing individuals from different localities may be warranted, since it may enhance genetic diversity in the breeding colony and wild populations, as shown in other endangered turtle studies (Nelson et al. 2024; Quinzin et al. 2019). Given the *S. bealei* breeding colony is relatively small (approximately 30 individuals) compared to other species (445 individuals in Çilingir et al. 2017; 136 individuals in Çilingir et al. 2019), has a slight deficiency (*F*_is_ = 0.181), and relatively low genetic diversity (current study; Lin et al. 2022; Shi et al. 2008), there is a risk of inbreeding. Using admixed individuals in reintroduction programs may become necessary in the future given the uncertain trajectories of many turtle populations across southern China.

Different populations may have been separated for sufficient time to develop adaptive differences for survival. Therefore, care should be taken when admixing the individuals since introducing non-native genotypes to existing wild populations can cause outbreeding depression (Frankham et al. 2011). Long-term genetic monitoring should be considered after reintroducing the admixed captive-bred individuals, as genetic changes may not be instantly apparent in a long-lived animal (Nelson et al. 2024). Various recommendations are encouraged for the post-reintroduction monitoring, such as conducting parentage analysis to ensure the captive-bred individuals are contributing to the next generation and evaluate the gene flow to ensure the wild populations are not overrepresented by the captive populations (IUCN/SSC 2013). For immediate breeding management, we have four recommendations based on IUCN/SSC (2013) guidelines. First is to separate pure Fujian individuals for breeding programs and population augmentation in Fujian. For breeding, to maintain genetic diversity and limit outbreeding depression, we suggest selecting pairs with relatively low co-ancestry (indicated by dark blue color, Fig. 6). If individuals are to be released, they should be quarantined to prevent potential disease/pathogen introduction. Second is to use Hong Kong pure individuals for local breeding programs, following the same general guidelines as the Fujian breeding group. However, since most breeding colony individuals are in the “Mixed” clade, effort should be made to find new individuals that fit in the HK1 and HK2 clades. Third is to maintain admixed individuals as a separate breeding group. If wild populations continue to decrease, and outbreeding depression and behavioral differences are unlikely, it may be prudent to release “Mixed” individuals to HK1 and HK2, and even mix Fujian and Hong Kong individuals. Our fourth recommendation is to conduct genetic monitoring of offspring. Differential survival of offspring may indicate genetic incompatibilities and pairs resulting in a high proportion of deaths should be stopped. As turtles are long-lived and genetic incompatibilities may take several generations to be apparent, Frankham et al. (2011) provide guidelines for helping predict outbreeding depression.

## Conclusion

In this study, we examined the population structure of the *S. bealei* between Fujian Province and Hong Kong. Three genetic clusters and the presence of admixed individuals were identified using ddRADseq data, which revealed more genetic structure compared to previous studies. To further understand the occurrence of admixed individuals, more wild samples from different localities across southern China are needed to better elucidate genetic clustering patterns. Additionally, our interpretation of genetic results would be strengthened by further ecological studies on the dispersal ability of *S. bealei* to better understand the likelihood of animal movement and natural admixture patterns.

We also inferred the geographic origin of individuals from an ex situ breeding colony based in Hong Kong. We emphasize that wild samples from a broader geographical range will be necessary to increase precision in inferring the geographic origin for individuals in the ex situ breeding colony, since many individuals are still of unknown provenance. Identifying the geographic origin of captive individuals is an important step for ex situ conservation. This study provides the first comprehensive genetic assessment of captive *S. bealei* breeding stock, directly informing current conservation breeding programs and enabling evidence-based breeding decisions.

## Supplementary Information

Below is the link to the electronic supplementary material.

Supplementary file1 (DOCX 672 KB)

## Data Availability

Raw demultiplexed sequences are available on the Sequence Read Archive (SRA) on the study accession number: PRJNA1113830.
