# Supplementary material for: Population genomics identifies genetic structure and admixture in the endangered Beale’s Eyed Turtle (Sacalia bealei), and implications for aquatic ecology and ex situ breeding
Source: Mar Life Sci Technol. 2026 Mar 3;8(2):446–58. doi: 10.1007/s42995-025-00340-7 (PMC13198569; doi:10.1007/s42995-025-00340-7)

**Supplementary Information**

**Population genomics identifies genetic structure and admixture in the endangered Beale’s eyed turtle (*Sacalia bealei*), and implications for aquatic ecology and *ex situ* breeding**

Wing-Him Lee^1^, Yik-Hei Sung^1,2^, Bi Wei Low^1,3*^, Jonathan J. Fong^1*^

^1^ Division of Science, Wu Jieh Yee School of Interdisciplinary Studies, Lingnan University, Hong Kong SAR 999077, China

^2^ School of Health, Sciences and Society, University of Suffolk, 19 Neptune Quay, Ipswich, IP4 IQJ, UK

^3^ Department of Biological Sciences, National University of Singapore, 16 Science Drive 4, Singapore 117558

* Corresponding authors:

Bi Wei Low: [biweilow@gmail.com](mailto:biweilow@gmail.com), +65-9023-3568

Jonathan J. Fong: [jonfong@ln.edu.hk](mailto:jonfong@ln.edu.hk), +852-2616-7081

**Supplementary** **Table S1.** *Sacalia bealei* samples of a subset of 42 individuals: detailed description of sampling localities and ddRADseq experiment results.

| **Sample ID** | **Locality** | **Reads** | **Coverage** |
| --- | --- | --- | --- |
| Fujian_1 | Fujian | 1854736 | 37.463 |
| Fujian_10 | Fujian | 1336907 | 31.374 |
| Fujian_11 | Fujian | 1475838 | 36.924 |
| Fujian_12 | Fujian | 1537228 | 43.18 |
| Fujian_13 | Fujian | 1026259 | 30.24 |
| Fujian_15 | Fujian | 595944 | 19.831 |
| Fujian_17 | Fujian | 3721067 | 83.282 |
| Fujian_19 | Fujian | 1661828 | 25.288 |
| Fujian_3 | Fujian | 969753 | 23.639 |
| Fujian_4 | Fujian | 4069205 | 77.581 |
| Fujian_5 | Fujian | 1689648 | 34.812 |
| Fujian_6 | Fujian | 2560076 | 54.803 |
| Fujian_7 | Fujian | 1390727 | 29.69 |
| HK1_15 | HK1 | 695653 | 20.446 |
| HK1_19 | HK1 | 718473 | 21.744 |
| HK1_30 | HK1 | 1733906 | 40.268 |
| HK1_37 | HK1 | 1932147 | 38.601 |
| HK1_40 | HK1 | 2022953 | 37.431 |
| HK1_43 | HK1 | 2799224 | 51.151 |
| HK1_44 | HK1 | 2739838 | 48.645 |
| HK1_45 | HK1 | 2421396 | 48.017 |
| HK1_52 | HK1 | 2524962 | 54.084 |
| HK1_53 | HK1 | 1939106 | 37.717 |
| HK1_54 | HK1 | 3175960 | 62.447 |
| HK1_55 | HK1 | 2015933 | 42.926 |
| HK1_56 | HK1 | 2395118 | 50.404 |
| HK1_61 | HK1 | 1468696 | 30.582 |
| HK1_65 | HK1 | 2550138 | 50.314 |
| HK1_71 | HK1 | 1188981 | 28.912 |
| HK2_10 | HK2 | 2341777 | 44.831 |
| HK2_11 | HK2 | 1429723 | 27.049 |
| HK2_12 | HK2 | 2029055 | 42.596 |
| HK2_2 | HK2 | 1782856 | 38 |
| HK2_3 | HK2 | 1418388 | 29.233 |
| HK2_41 | HK2 | 1627597 | 33.19 |
| HK2_50 | HK2 | 2344311 | 43.882 |
| HK2_6 | HK2 | 1682622 | 37.014 |
| HK2_68 | HK2 | 2334402 | 44.477 |
| HK2_69 | HK2 | 1524825 | 33.33 |
| HK2_7 | HK2 | 2000941 | 41.269 |
| HK2_70 | HK2 | 1240754 | 28.542 |
| HK2_8 | HK2 | 1838214 | 38.676 |

**Supplementary Table S2.** *Sacalia bealei* samples of a complete set of 75 individuals: detailed description of sampling localities and ddRADseq experiment results.

| **Sample ID** | **Locality** | **Reads** | **Coverage** |
| --- | --- | --- | --- |
| Fujian_1 | Fujian | 1776437 | 40.16 |
| Fujian_10 | Fujian | 1278527 | 33.654 |
| Fujian_11 | Fujian | 1424483 | 37.939 |
| Fujian_12 | Fujian | 1494143 | 44.661 |
| Fujian_13 | Fujian | 980909 | 30.623 |
| Fujian_15 | Fujian | 568438 | 19.939 |
| Fujian_17 | Fujian | 3506678 | 86.021 |
| Fujian_19 | Fujian | 1590487 | 26.066 |
| Fujian_3 | Fujian | 919169 | 25.052 |
| Fujian_4 | Fujian | 3879567 | 84.202 |
| Fujian_5 | Fujian | 1592577 | 37.112 |
| Fujian_6 | Fujian | 2415386 | 58.897 |
| Fujian_7 | Fujian | 1302033 | 31.604 |
| HK1_15 | HK1 | 662273 | 20.379 |
| HK1_19 | HK1 | 685240 | 21.649 |
| HK1_30 | HK1 | 1656919 | 40.94 |
| HK1_37 | HK1 | 1849497 | 40.662 |
| HK1_40 | HK1 | 1943530 | 39.407 |
| HK1_43 | HK1 | 2684570 | 54.134 |
| HK1_44 | HK1 | 2622744 | 51.18 |
| HK1_45 | HK1 | 2321301 | 50.721 |
| HK1_52 | HK1 | 2425721 | 57.001 |
| HK1_53 | HK1 | 1856135 | 39.563 |
| HK1_54 | HK1 | 3038262 | 65.743 |
| HK1_55 | HK1 | 1928277 | 45.121 |
| HK1_56 | HK1 | 2294758 | 52.977 |
| HK1_61 | HK1 | 1405841 | 32.166 |
| HK1_65 | HK1 | 2441552 | 52.901 |
| HK1_71 | HK1 | 1136649 | 30.238 |
| HK2_10 | HK2 | 2249274 | 47.363 |
| HK2_11 | HK2 | 1368424 | 28.382 |
| HK2_12 | HK2 | 1942941 | 44.751 |
| HK2_2 | HK2 | 1704240 | 39.951 |
| HK2_3 | HK2 | 1350186 | 30.524 |
| HK2_41 | HK2 | 1549848 | 34.869 |
| HK2_50 | HK2 | 2236217 | 46.298 |
| HK2_6 | HK2 | 1612617 | 38.91 |
| HK2_68 | HK2 | 2224201 | 46.841 |
| HK2_69 | HK2 | 1453191 | 34.88 |
| HK2_7 | HK2 | 1912457 | 43.484 |
| HK2_70 | HK2 | 1181168 | 29.87 |
| HK2_8 | HK2 | 1757840 | 40.704 |
| HKHERP_10 | Unknown | 1155221 | 28.428 |
| HKHERP_12 | Unknown | 962198 | 25.884 |
| HKHERP_15 | Unknown | 1691049 | 40.532 |
| HKHERP_16 | Unknown | 628879 | 19.096 |
| HKHERP_2 | Unknown | 605338 | 17.163 |
| HKHERP_20 | Unknown | 791455 | 21.312 |
| HKHERP_21 | Unknown | 1391829 | 38.298 |
| HKHERP_22 | Unknown | 581788 | 16.708 |
| HKHERP_23 | Unknown | 1099872 | 28.099 |
| HKHERP_25 | Unknown | 827349 | 19.838 |
| HKHERP_26 | Unknown | 1651301 | 37.617 |
| HKHERP_27 | Unknown | 1555976 | 39.248 |
| HKHERP_29 | Unknown | 601382 | 19.399 |
| HKHERP_3 | Unknown | 1858457 | 40.812 |
| HKHERP_32 | Unknown | 721239 | 23.003 |
| HKHERP_33 | Unknown | 728430 | 19.995 |
| HKHERP_34 | Unknown | 663519 | 21.162 |
| HKHERP_36 | Unknown | 1027917 | 27.462 |
| HKHERP_37 | Unknown | 1128714 | 29.168 |
| HKHERP_39 | Unknown | 1016984 | 27.02 |
| HKHERP_4 | Unknown | 1022890 | 25.534 |
| HKHERP_45 | Unknown | 1043403 | 30.216 |
| HKHERP_46 | Unknown | 1537078 | 42.137 |
| HKHERP_47 | Unknown | 1228343 | 32.726 |
| HKHERP_48 | Unknown | 1067020 | 27.19 |
| HKHERP_49 | Unknown | 1134563 | 29.385 |
| HKHERP_5 | Unknown | 1362206 | 33.346 |
| HKHERP_50 | Unknown | 565619 | 15.025 |
| HKHERP_51 | Unknown | 1546601 | 40.285 |
| HKHERP_53 | Unknown | 1144801 | 29.093 |
| HKHERP_6 | Unknown | 756903 | 20.772 |
| HKHERP_7 | Unknown | 1044187 | 28.396 |
| HKHERP_8 | Unknown | 1141974 | 26.281 |

**Supplementary Table S3.** *P*-value for all pairs of localities

|  | HK1 | HK2 | Fujian |
| --- | --- | --- | --- |
| HK1 | -- |  |  |
| HK2 | 0.01 | -- |  |
| Fujian | 0.01 | 0.01 | -- |

**Supplementary Fig. S1.** The following principal component analysis (PCA) plots are generated using the 75-SingleSNP dataset with different parameter combinations in Stacks. Dataset was assembled with distance allowed between stacks (M) = 3 and distance between catalogue loci (n) = 3 for denovo_map.pl pipeline (Supplementary Fig. S1A). The analyses presented in the main text correspond to the parameter combination with distance allowed between stacks (M) = 5 and distance between catalogue loci (n) = 5 for denovo_map.pl pipeline (Supplementary Fig. S1B).

| A | B |
| --- | --- |
| **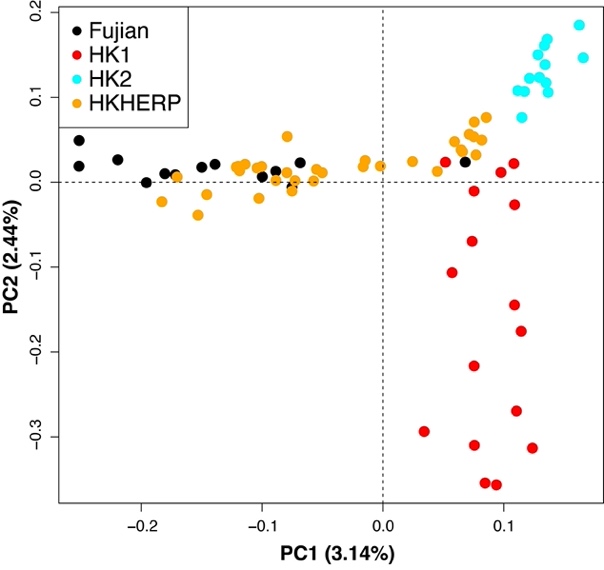** | **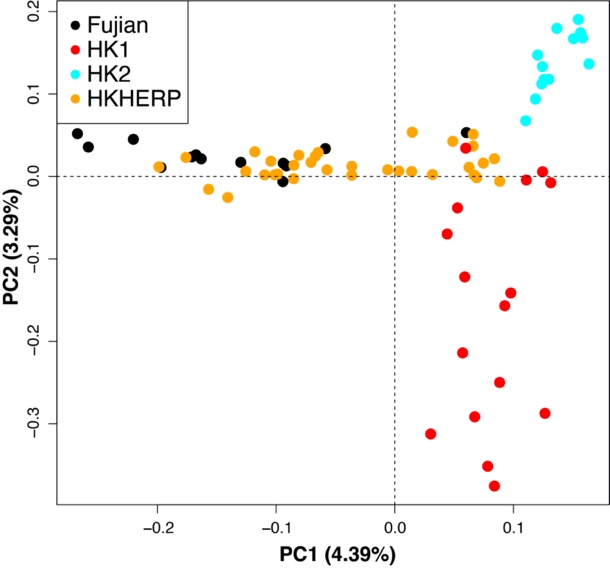** |

**Supplementary Fig. S2.** ΔK calculated using the Evanno method in CLUMPAK (42-SingleSNP).


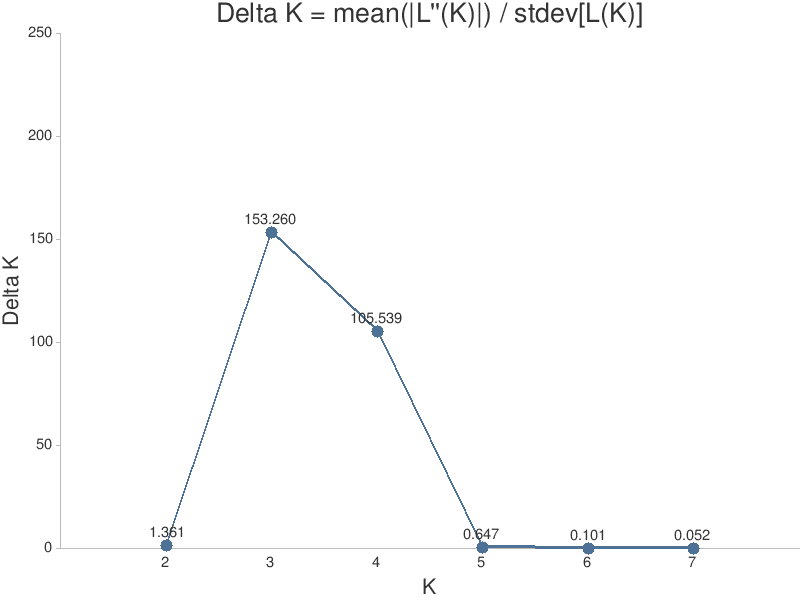


**Supplementary Fig. S3.** ΔK calculated using the Evanno method in CLUMPAK (75-SingleSNP).


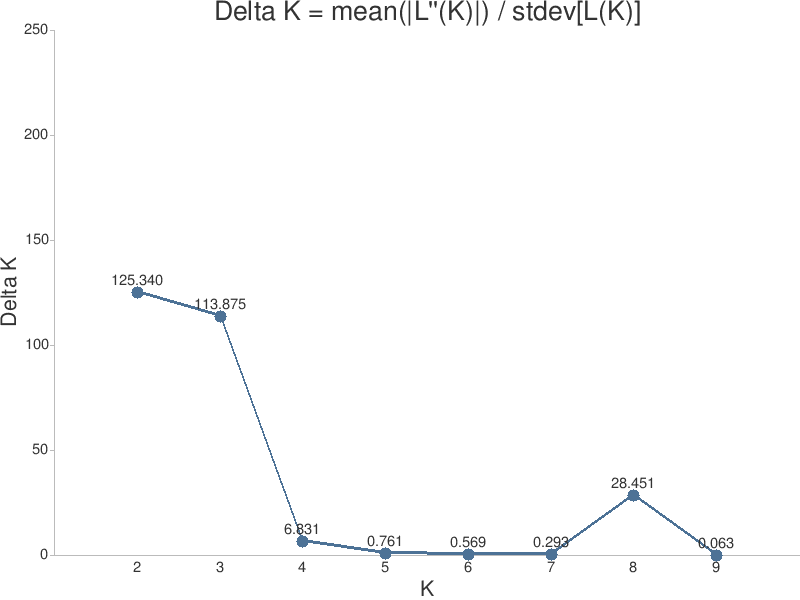


**Supplementary Fig. S4.** Hybrid detection power (*i.e.*, accuracy x efficiency) across different critical posterior probability thresholds based on two replicates of three simulated datasets of multigenerational hybrids, with Hong Kong (HK1 and HK2 combined) and Fujian genotypes designated as Pure1 and Pure2 parental populations, respectively. Plots are faceted by the size of diagnostic SNP panels (100, 200, 300 or 400 highest *F*_ST_ and low-linkage SNPs). Solid lines represent means whereas dashed lines are standard deviations.


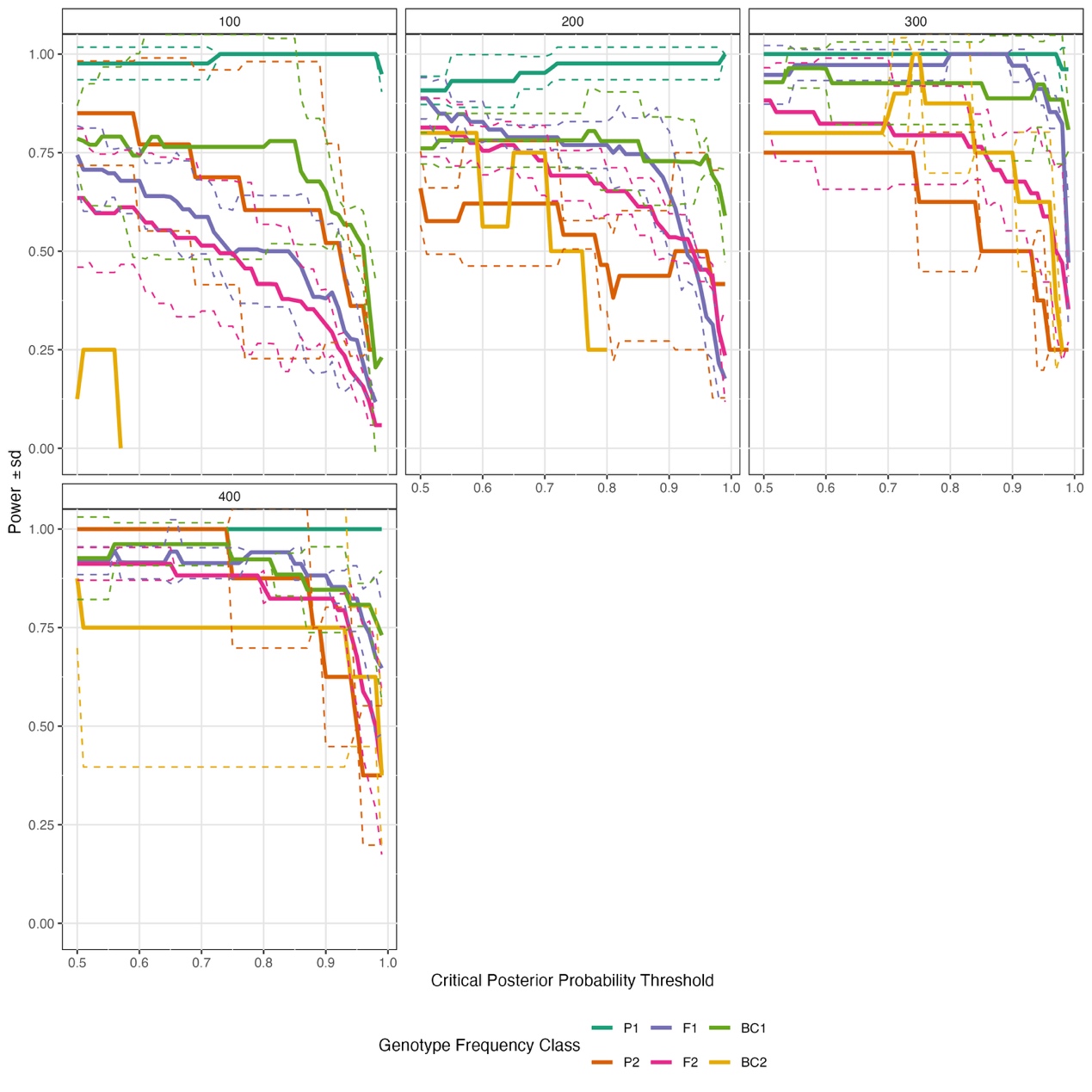


**Supplementary Fig. S5.** Hybrid detection power (*i.e.*, accuracy x efficiency) across different critical posterior probability thresholds based on two replicates of three simulated datasets of multigenerational hybrids, with HK1 and HK2 genotypes designated as Pure1 and Pure2 parental populations, respectively. Plots are faceted by the size of diagnostic SNP panels (100, 200, 300 or 400 highest *F*_ST_ and low-linkage SNPs). Solid lines represent means whereas dashed lines are standard deviations.


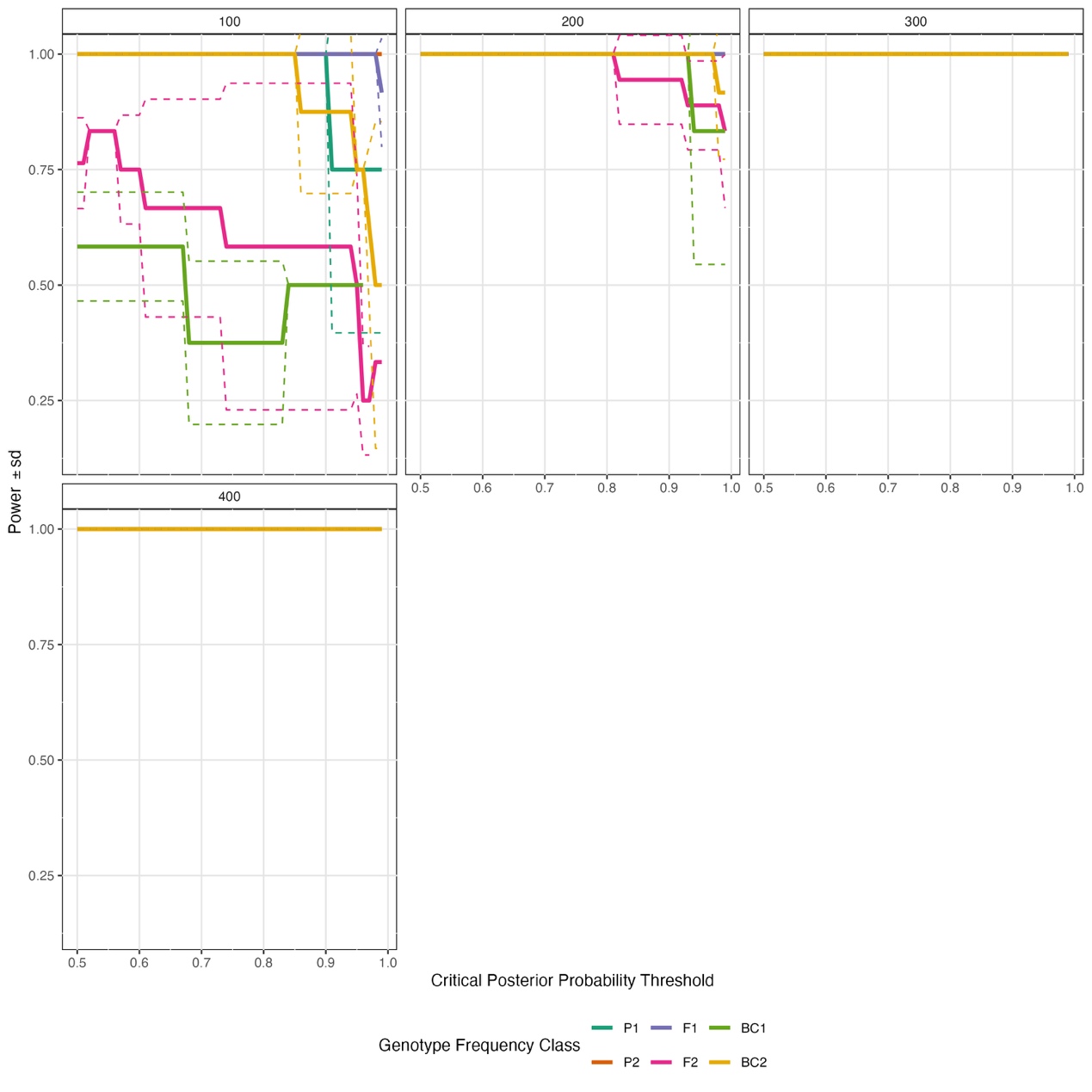

Supplement: Supplementary file 1 — Supplementary file1 (DOCX 672 KB) [file 42995_2025_340_MOESM1_ESM.docx]
